# Examination of short and long term complications of thermocautery, plastic clamping, and surgical circumcision techniques

**DOI:** 10.12669/pjms.343.15273

**Published:** 2018

**Authors:** Levend Ozkan

This refers to the manuscript entitled “Examination of short and long term complications of thermocautery, plastic clamping, and surgical circumcision techniques” published in November-December 2017 issue of Pakistan Journal of Medical Sciences.^1^

This is a remarkable study despite the limitations pointed out by the author. I think that “thermocautery” should be considered as a device rather than a circumcision technique and “thermocautery - assisted shield / or guillotine technique” would be a more proper definition. In fact, this is a modification of a standard and common surgical circumcision technique, where the skin is cut with thermocautery instead of scalpel or scissor. It is also very effective for coagulating small vessels and obviates the need for electrosurgical cautery.

Thermocautery is commonly mistaken for monopolar electrocautery, even by urology specialists and pediatric surgeons. The working principle of the devices is completely different. It is well known that monopolar electrocautery should be used very carefully in penile surgery -especially in children - due to the risk of electrical current damage. However thermocautery can be safely used over a metal clamp and direct contact to the clamp is harmless.

Thermocautery-assisted shield technique is being used for over 30 years at our circumcision clinic (Kemal Ozkan Circumcision Clinic) and over 80.000 circumcisions have been performed without any complication related to the device. Considering that a very large percentage of the circumcisions are being performed under local anesthesia in our country, reduced procedure time and bloodless operating scene are the major advantages of this method. Without any doubt, experience and precision are the key factors regardless of the surgical technique.
